# Atomic Resolution Structure of a Protein Prepared by Non-Enzymatic His-Tag Removal. Crystallographic and NMR Study of *Gm*SPI-2 Inhibitor

**DOI:** 10.1371/journal.pone.0106936

**Published:** 2014-09-18

**Authors:** Edyta Kopera, Wojciech Bal, Martina Lenarčič Živkovič, Angela Dvornyk, Barbara Kludkiewicz, Krystyna Grzelak, Igor Zhukov, Włodzimierz Zagórski-Ostoja, Mariusz Jaskolski, Szymon Krzywda

**Affiliations:** 1 Institute of Biochemistry and Biophysics, Polish Academy of Sciences, Warsaw, Poland; 2 Slovenian NMR Centre, National Institute of Chemistry, Ljubljana, Slovenia; 3 NanoBioMedical Centre, A. Mickiewicz University, Poznan, Poland; 4 Department of Crystallography, Faculty of Chemistry, A. Mickiewicz University, Poznan, Poland; 5 Center for Biocrystallographic Research, Institute of Bioorganic Chemistry, Polish Academy of Sciences, Poznan, Poland; NCI-Frederick, United States of America

## Abstract

Purification of suitable quantity of homogenous protein is very often the bottleneck in protein structural studies. Overexpression of a desired gene and attachment of enzymatically cleavable affinity tags to the protein of interest made a breakthrough in this field. Here we describe the structure of *Galleria mellonella* silk proteinase inhibitor 2 (*Gm*SPI-2) determined both by X-ray diffraction and NMR spectroscopy methods. *Gm*SPI-2 was purified using a new method consisting in non-enzymatic His-tag removal based on a highly specific peptide bond cleavage reaction assisted by Ni(II) ions. The X-ray crystal structure of *Gm*SPI-2 was refined against diffraction data extending to 0.98 Å resolution measured at 100 K using synchrotron radiation. Anisotropic refinement with the removal of stereochemical restraints for the well-ordered parts of the structure converged with *R* factor of 10.57% and *R*_free_ of 12.91%. The 3D structure of *Gm*SPI-2 protein in solution was solved on the basis of 503 distance constraints, 10 hydrogen bonds and 26 torsion angle restraints. It exhibits good geometry and side-chain packing parameters. The models of the protein structure obtained by X-ray diffraction and NMR spectroscopy are very similar to each other and reveal the same β_2_αβ fold characteristic for Kazal-family serine proteinase inhibitors.

## Introduction

Despite significant methodological progress [1], structural studies of proteins still require significant amount of pure samples. To achieve this goal, the affinity tag methodology is commonly used. However, the presence of an affinity tag may affect the biological activity of a target protein and interfere with crystallization [2]. Therefore it is recommended to remove affinity tags from the purified protein. This has often been, however, the Achilles heel of this approach. The proteinase-mediated enzymatic cleavage commonly used for affinity tag removal poses serious risks, such as non-specific degradation of the target protein [3]–[5]. Moreover, costly preparative-scale purification of the cleavage products is necessary, including the proteinase inactivation and removal step. Chemical cleavage agents are suggested as inexpensive alternative to proteolytic enzymes [6]–[8]. However, none of them is commonly used due to their low specificity and harsh reaction conditions [9]–[11].

Our previous studies demonstrated that Ni(II) ions hydrolyze the peptide bond preceding the serine or threonine residue in (S/T)XHZ peptide sequences [12]. The specificity of the cleavage was confirmed for a range of peptides and the reaction mechanism was precisely elucidated [13]. Recently we have positively verified the biotechnological applicability of the Ni(II)-depended peptide bond cleavage reaction for the recombinant *Gm*SPI-2 protein, which is the subject of our structural analysis in this work. The protein purification procedures in that study were performed on an analytical scale [14]. However those results indicated that the methodology could be easily scaled up for preparative purification of recombinant proteins for structural studies.

**Figure 1:**
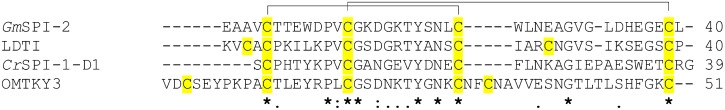
Sequence alignment of classical (OMTKY3), non-classical group 1 (*Cr*SPI-1-D1), non-classical group 2 (LDTI) and *Gm*SPI-2 Kazal-family serine proteinase inhibitors. The alignment was calculated with *ClustalW2* [46]. Cysteine residues are highlighted in yellow. Amino acids identical in all four proteins are marked with an asterisk (*), conservative substitutions with a colon (:), and semi-conservative substitutions with a period (.).

The *Gm*SPI-2 protein is a structurally unique Kazal-family serine proteinase inhibitor identified in the silk of wax moth *Galleria mellonella* [15]. It is the shortest Kazal-family serine proteinase inhibitor in animals. Unlike most Kazal-family serine proteinase inhibitors, where each functional domain consists of 50–60 amino acid residues with six conserved cysteines, *Gm*SPI-2 is a single domain inhibitor of 36 residues with only four cysteines (Fig. 1). Computer modeling suggested that, in contrast to typical Kazal-family serine proteinase inhibitors, the conformation of *Gm*SPI-2 includes not three but only two loops which are stabilized and closed into rings by disulfide bridges between the four conserved cysteines [15]. The inhibitor exhibits high activity against subtilisin and proteinase K (proteases from *Bacillus subtilis* and the *Tritirachium album*, respectively) [15]. Recombinant *Gm*SPI-2 activity is identical with the native protein [16]. Since *Gm*SPI-2 is a much potent proteinase inhibitor than some commercially available inhibitors (*e.g.* AEBSF, 4-(2-aminoethyl) benzenesulfonyl fluoride hydrochloride; [17]), it could be used as a replacement or supplement of available inhibitors or inhibitor cocktails. Additionally, when fused to a target protein, *Gm*SPI-2 could protect the target protein against proteinase degradation [17], [18]. Thus, *Gm*SPI-2 can be considered as a valuable and economically important protective tool in biotechnology for enhancing the yields and prolonging the life of desired protein products.

Here we discuss the application of the previously described nickel-based purification methodology, scaled up for this structural work, and demonstrate the usefulness of this innovative approach for structural studies. The determinations of the atomic resolution X-ray and high quality NMR structure of the *Gm*SPI-2 protein, both critically dependent on large quantities of highly pure protein samples, were possible partially because of this protein purification method.

## Materials and Methods

### *Gm*SPI-2-SRHWAP-H_6_ fusion protein expression and purification

The cDNA sequence encoding SPI-2 protein with modified C-terminal end was used as a template (Leu codon was added as described [16]). The primers were extended to introduce a PstI restriction site at the 5′ end of the amplified product and an XbaI restriction site at the 3′ end, followed nucleotides encoding SRHWAP and six histidyl residues. The alternative SPI2-SRHWAP-H_6_ fusion protein was designed in order to improve the yield of purification and the purity of the final product. The appropriate gene construct was successfully cloned under the control of AOX promoter in a pPICZαB vector (Invitrogen), using standard methods. As a result of the cloning procedure and the pre-protein processing in *Pichia pastoris*, *Gm*SPI-2 was extended by the GluAlaAla- tripeptide at the N-terminus and by the -Leu40 residue at the C-terminus. The fusion protein secreted to the media was initially purified by affinity chromatography on Ni-NTA-agarose (Qiagen) in the presence of 20 mM phosphate buffer pH 7.4, containing 0.5 M NaCl. The fusion protein was then eluted from the column with 250 mM imidazole and dialyzed overnight against water in order to remove the excess of salts. Typically 2 ml of elution fraction was dialyzed against 2 L of water. Next, the protein was purified by HPLC on a Vydac C18 semipreparative column. The eluting solvent A was 0.1% TFA/water and solvent B was 0.1% TFA/90% acetonitrile/water. A linear gradient of acetonitrile from 10% to 40% in 30 min was applied at a flow rate of 2 mL/min, with detection at 220 nm and 280 nm. After elution, the fusion protein was frozen and lyophilized. The HPLC purification step was applied to assess the amount of *Gm*SPI-2.

### Affinity tag cleavage

The *Gm*SPI-2 fusion protein after lyophilization was weighed (portions of 5–7 mg) and dissolved in 20 mM phosphate buffer pH 7.4, containing 0.5 M NaCl and incubated with Ni-NTA-agarose (Qiagen) for 2 h at 4°C. Then the *Gm*SPI-2 fusion protein immobilized on Ni-NTA agarose was incubated in 100 mM Hepes buffer pH 8.2, containing 150 mM NaCl and 7.5 Mm NiCl_2_ at 50°C for 19 h. The *Gm*SPI-2 protein obtained in the flow-through fraction was further purified using the Breeze HPLC system (Waters) on a Vydac C18 semipreparative column. The eluting solvent A was 0.1% TFA/water and solvent B was 0.1% TFA/90% acetonitrile/water. A linear gradient of solvent B from 10% to 40% in 30 min was used at a flow rate of 2 mL/min. The molecular mass of the collected HPLC single peak was measured on a Q-Tof1 ESI MS spectrometer (Micromass).

### Crystallization

Screening for crystallization conditions was performed manually using Crystal Screen and Crystal Screen 2 [19] and the hanging-drop vapor-diffusion technique at 292 K, by mixing 1 µl protein (6.5 mg ml^−1^ in water) and 1 µl reservoir solution. Needle-like crystals grew to dimensions of 0.6×0.05×0.05 mm within one week over a reservoir solution consisting of 1.4 M sodium citrate and 0.1 M Hepes pH 7.5 (Crystal Screen condition no. 38). For cryoprotection, the crystal was transferred to a solution consisting of the reservoir solution supplemented with 20% (v/v) glycerol.

### X-Ray data collection

Diffraction data were measured at 100 K on a Rayonics MX-225 CCD detector at beamline BL 14.1 of the Berliner Elektronenspeicherring-Gesellschaft für Synchrotronstrahlung m.b.H. (BESSY, Berlin). Integration, scaling and merging of the intensity data was accomplished using the *XDS* package [20]. The best crystal diffracted to 0.95 Å but due to a glitch of the data collection program, only 52° of the high-resolution pass were collected. Therefore, the low-resolution and truncated high-resolution passes were scaled together with the data collected for another crystal. This gave the complete data set at 0.98 Å, characterized in Table 1. An overall *B*-factor of 8.2 A^2^ was estimated from the Wilson plot using the *XSCALE* program from *XDS* package [20].

**Table 1:** X-ray data collection and model refinement statistics.

Data Collection	
Crystal size (mm)	0.7×0.05×0.05
Space group	*P*2_1_2_1_2_1_
Unit cell parameters (Å)	*a* = 27.27, *b* = 31.24, *c* = 35.74
X-ray source	BESSY 14.1
Temperature (K)	100
No. of images	360^a^/180/52
Oscillation angle (°)	1.0^a^/2.0/1.0
Wavelength (Å)	0.91841^a^/0.80000/0.80000
Resolution (Å)	20.00–0.98 (1.01–0.98)^b^
* R*_int_ c(%)	7.2 (46.0)
<*I*/σ*I*>	22.52 (2.04)
Reflections	
measured	222092
unique	17179
in test set	1031
Mosaicity (°)	0.13
Completeness (%)	94.8 (84.5)
Redundancy	12.9 (2.3)
Wilson *B*-factor (Å^2^)	8.2
**Refinement**	
Resolution (Å)	20.00–0.98 (1.02–0.98)
No. reflections	17179 (1790)
* R*_work_/*R*_free_ d	10.57/12.91
No. of atoms	
protein	328
solvent	96
* B*-factors (Å^2^)	
protein	6.5
solvent	12.1
Rmsd from ideal	
bond lengths (Å)	0.023
angle distances (Å)	0.058
Ramachandran statistics (%)	
favored	90.6
additional	9.4
PDB code	4hgu

a Values for crystal no. 1.

b Values in parentheses are for the highest resolution shell.

c *R*_int_ = Σ_hkl_Σ_i_|*I_i_*(*hkl*)−<*I*(*hkl*)>|/Σ_hkl_Σ_i_ *I*_i_(*hkl*), where *I*_i_(*hkl*) is the *i*th measurement of the intensity of reflection *hkl* and <*I*(*hkl*)> is the mean intensity of reflection *hkl*.

d *R* = Σ||*F*_o_|−|*F*_c_||/Σ|*F*_o_|, where *F*_o_ and *F*_c_ are the observed and calculated structure factor amplitudes, respectively.

Data measured to high resolution with a synchrotron X-ray beam can be contaminated with effects of crystal radiation damage. However, the plot of decay *R* factor [21] against frame-number difference was around zero and scaling factors for the individual diffraction images fluctuated around 1 without any decreasing trend, indicating no or very little effect of radiation damage. The data were also checked for diffraction anisotropy [22]. A very low spread in values of the three principal components (0.48 Å^2^) indicated almost no anisotropy.

### X-Ray structure solution and refinement

The structure was solved by molecular replacement using the *MOLREP* program from the *CCP4* suite [23], [24] and the structure of leech-derived tryptase inhibitor (LDTI; PDB code 1an1; [25]) as the search model. The amino-acid sequence of the model shares 40% identity and 60% similarity (*LALIGN*; [26]) with *Gm*SPI-2. The initial maximum-likelihood structure-factor refinement was carried out in *REFMAC* [27] using all data, with the exception of 1031 reflections (6%) flagged for cross-validation purposes. No σ cutoff was applied. The manual rebuilding of the model was performed in *COOT* [28]. The conjugate-gradient least-squares refinement in *SHELXL* [29] was used to refine the model at the later stages. The main steps of the refinement included (1) isotropic refinement with manually added water molecules and sodium ions, (2) anisotropic refinement, (3) addition of H atoms according to geometrical criteria implemented in *SHELXL*, (4) refinement of the occupancies of partially occupied/alternate conformations and solvent atoms, and finally (5) removal of the restraints for the well-ordered parts of the model. Six side-chains, namely Glu1, 8 and 38, Val4, Asp10 and Leu23, as well as two C_α_ atoms, of Val4 and Glu38, were modeled with alternate conformations. Additionally, the C_γ_, C_δ_, O_ε1_ and O_ε2_ atoms of Glu36, and the C_ε_ and N_ζ_ atoms of Lys15 and Lys18 were given partial occupancies. The stereochemical restraints were retained throughout refinement only for these side chains/atoms.

Sodium ions were found in the electron density map and identified on the basis of coordination number (6) and Na^+^ ⋅ ⋅ ⋅ O distances (2.33–2.58 Å), in agreement with the high concentration of sodium cations in the crystallization solution (1.4 M sodium citrate). In the final round, all data were used in the refinement, including the *R_free_* reflections, leading to the convergence with *R* values of 8.62% for the 14133 reflections with *F*_o_>4σ(*F*_o_) and 10.57% for all 17179 reflections (Table 1).

At the end of the refinement, one cycle of full-matrix minimization was calculated with all stereochemical restraints removed and with all parameter shifts damped to zero, which permitted the estimation of the standard uncertainties (s.u.) in all positional parameters. Full refinement statistics are given in Table 1.

### NMR resonance assignment and structure determination

All NMR experiments were performed using an 18.8 T Varian DirectDrive 800 NMR spectrometer (operating ^1^H frequency 799.811 MHz). The NMR sample was obtained by diluting a *Gm*SPI-2 protein sample in 90%/10% H_2_O/D_2_O, 20 mM phosphate buffer pH 4.5, with 50 mM NaCl, to a final concentration of 0.5 mM. Assignments of ^1^H, ^13^C, and ^15^N resonances were achieved utilizing standard methods on the basis of 2D TOCSY and NOESY data [30]. The homonuclear experiments were supplemented with 2D heteronuclear ^1^H-^15^N and ^1^H-^13^C HSQC spectra acquired at natural abundance of ^15^N and ^13^C nuclei. All NMR spectra were referenced using external DSS (sodium 2,2′-dimethyl-2-silapentane-5-sulfonate) [31] and processed by *NMRPipe* software [32]. The three-dimensional structure of the *Gm*SPI-2 protein in solution was solved by standard 2D NMR techniques on the basis of 503 (141 intra-residue, 138 sequential, 84 medium, and 140 long range) distance constraints provided by the analysis of 2D homonuclear ^1^H-^1^H NOESY spectra acquired with 120 ms mixing time. 26 Restraints for the backbone φ and ψ torsion angles were defined using the analysis of chemical shifts with the program *TALOS+* [33]. This procedure provided 58 restraints for the φ and ψ torsion angles for 29 residues, which were predicted as ‘good’ by *TALOS+*. Additionally, 20 distance constraints for 10 hydrogen bonds were defined as r*_HN-O_* = 1.5–2.8 and r*_N-O_* = 2.4–3.5 Å (Table 2). 200 structures were calculated by the *CYANA* (version 3.0) software [34]. Finally, 17 conformers, selected on the basis of low target function criteria, were subjected to a refinement procedure in a water shell using the *YASARA* program suite [35]. The statistics of NOE distance restraints together with the analysis of the ensemble of 17 lower energy structures evaluated on the basis of NMR data are presented in Table 2.

**Table 2:** NMR restraints and structural statistics for the ensemble of 17 lower energy of *Gm*SPI-2 conformers.

NOESY distance restraints^a^	506
intraresidual (|*i-j*| = 0)	135
sequential (|*i-j*| = 1)	135
medium-range (1<|*i-j*| ≤5)	77
long-range (|*i-j*|>5)	135
Hydrogen bonds	24
Constraints per residue	13.3
Torsion angle restraints	
backbone (φ/ψ)	26
Structure Z-scores	
1-st generation packing quality	−0.125±1.034
2-nd generation packing quality	4.836±1.836
Ramachandran plot appearance	−0.923±0.405
χ1/χ2 rotamer normality	−2.538±0.874
Backbone conformation	0.189±0.470
RMS Z-scores^b^	
bond lengths	1.174±0.008
bond angles	0.513±0.021
omega angle restraints	0.929±0.115
side chain planarity	0.673±0.117
improper dihedral distribution	0.878±0.099
Ramachandran statistics^c^ (%)	
favored	86.9
additional	13.1
Rmsd from the mean structure	
backbone atoms (Å)	0.63±0.27
heavy atoms (Å)	0.96±0.20
PDB code	2m5x
number of models	17

a None of the 17 structures has a distance violation of more than 0.2 Å and a dihedral angle violation more than 5°.

b The quality of the ensemble of 17 lowest-energy structures was checked by *PROCHECK-NMR*, version 3.4 [48].

c The ensemble of structures was validated by the *WhatIf* program [49].

## Results and Discussion

### Purification of *Gm*SPI-2 protein for structural studies

**Figure 2:**
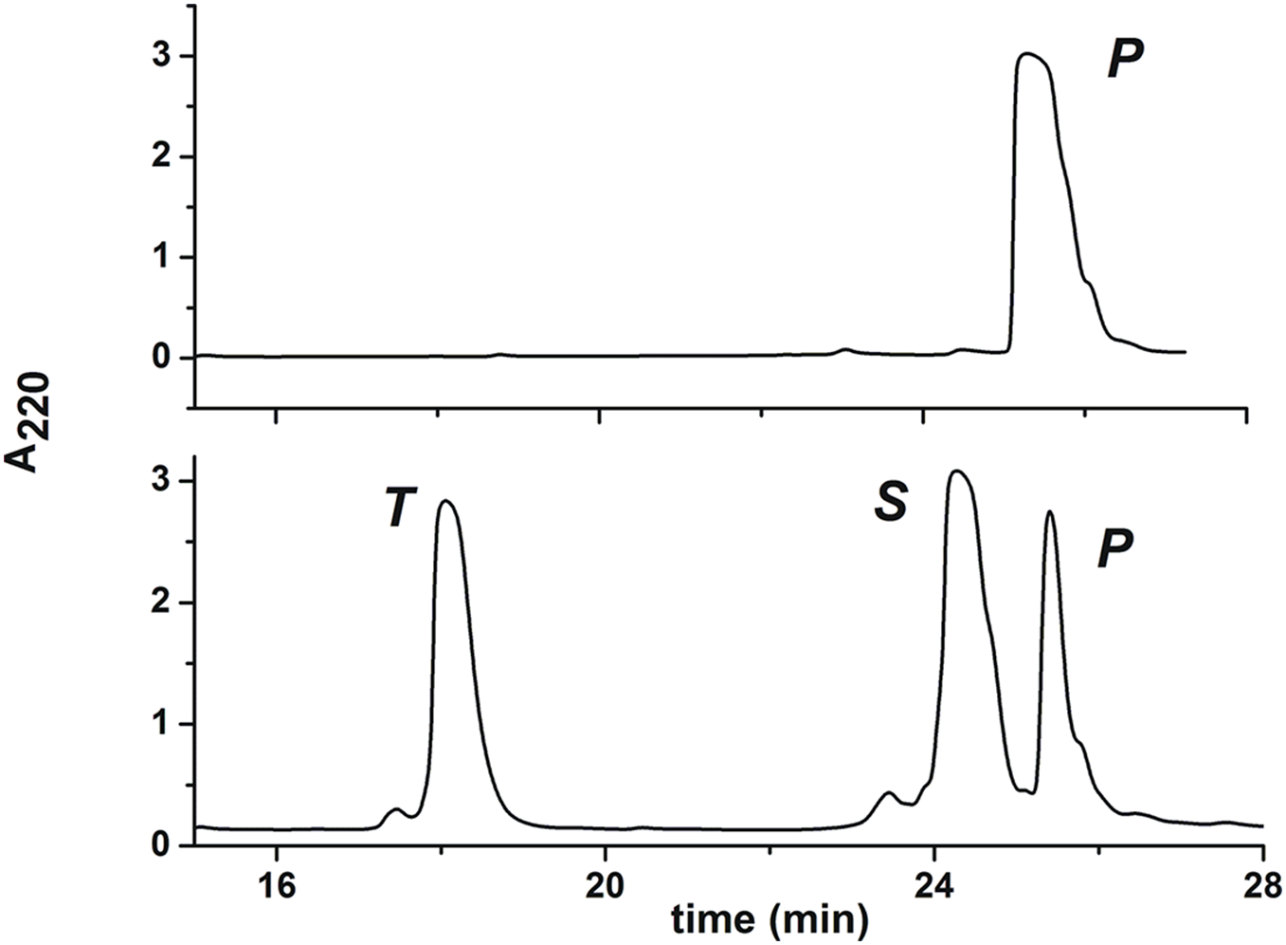
Preparative HPLC chromatograms of protein samples after 19 h of incubation at 50°C. Incubation buffer (top) and wash buffer (bottom). The peak labels denote the reaction substrate and products, identified using ESI-MS: P, pure *Gm*SPI-2 protein (4310 Da); T, the SRHWAP-H_6_ peptide (the extended His-tag, which is the C-terminal hydrolysis product, 1574 Da); S, substrate (fusion protein, 5868 Da).

The fusion construct designed for the affinity purification/tag removal approach according to our nickel-based method, contains the Ni(II)-specific SRHW sequence cloned between the *Gm*SPI-2 target protein and its C-terminal His-tag. Two additional amino acids, Ala and Pro, were added after the crucial tetrapeptide sequence as a spacer in order to avoid unwanted interactions between this sequence and the tag. The purification procedure of the *Gm*SPI-2-SRHWAP-H_6_ fusion protein produced in *Pichia pastoris* culture, included initial affinity chromatography on Ni-NTA agarose, followed by HPLC. The latter purification step was applied in order to assess the amount of the fusion protein for affinity tag removal, thus enabling a quantitative evaluation of this novel procedure at the preparative scale of 5–7 mg of protein. The purified *Gm*SPI-2-SRHWAP-H_6_ protein was then reloaded on the Ni-NTA agarose column, and incubated with excess of Ni(II) ions. The cleavage conditions were based on our recently published analytical-scale paper [14]. The flow-through fraction collected contained only the pure *Gm*SPI-2 protein, with the SRHWAP-H_6_ tag removed, as evidenced by the presence of a single peak on the HPLC chromatogram of this fraction (Fig. 2). A chromatogram of the wash buffer fraction indicated that a small amount of *Gm*SPI-2 got stuck to the agarose column. The molecular masses of the collected peaks confirmed the absence of unspecific cleavage. The total yield of pure *Gm*SPI-2 was 70% in repeated experiments. The efficiency of the cleavage reaction was calculated precisely using the values of HPLC peak areas corresponding to the substrate and products before and after protein incubation with Ni(II) ions.

### The quality of the crystal structure

The final crystallographic *Gm*SPI-2 model (PDB code 4hgu) consists of 328 protein-atom sites for 300 non-hydrogen atoms, 92 water molecules and 4 sodium ions. The electron density for all atoms of the main-chain and fully occupied atoms of side-chains is exceptionally good. The number of reflections per parameter in the final cycle of refinement was 4.5, sufficient to justify refinement without any stereochemical restraints for the well-ordered parts of the molecule. Despite this radical approach, 90.6% of the residues are located in the most favored regions and 9.4% in the additionally allowed regions of the Ramachandran plot [36]. This, together with very good refinement statistics, confirmed that this refinement approach was correct.

**Figure 3:**
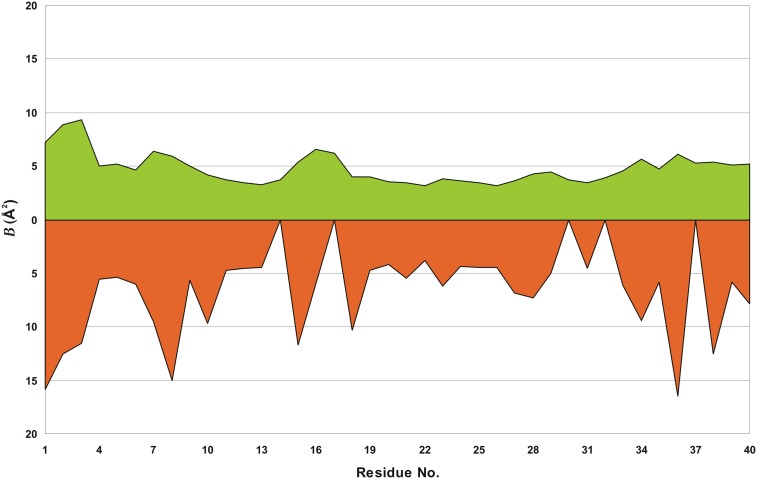
Plot of *B*_eq_ averaged over main-chain (green) and side-chain (orange) atoms of the crystallographic model. Values of zero in the lower plot correspond to glycines.

The average value of the atomic displacement parameter (*B*_eq_) for *Gm*SPI-2 is 6.5 Å^2^. The N/C termini in protein structures are very often disordered. The pattern of *B*_eq_ values along the polypeptide chain shows that the whole main chain is well ordered (Fig. 3). Especially the residues from 11 to 14 (forming β-strand 1) and from 19 to 33 (forming β-strand 2 and α-helix) have slightly lower than average *B*_eq_ value (with *B*_eq_ values of 3.53 and 3.75 Å^2^ for residues 11–14 and 19–33, respectively). The mean *B*_eq_ values of the main-chain, side-chain and solvent atoms of *Gm*SPI-2 are 4.8, 8.2 and 12.1 Å^2^, respectively. There are only two structures of Kazal-family serine proteinase inhibitors determined at a comparable resolution which are available in the PDB, namely 1r0r at 1.10 Å and 2 gkr at 1.17 Å. The corresponding values of *B*_eq_ for those structures are much higher, 16.7, 19.3 and 38.2 Å^2^ for 1r0r and 14.4, 17.0 and 32.6 Å^2^ for 2 gkr, respectively.

**Figure 4:**
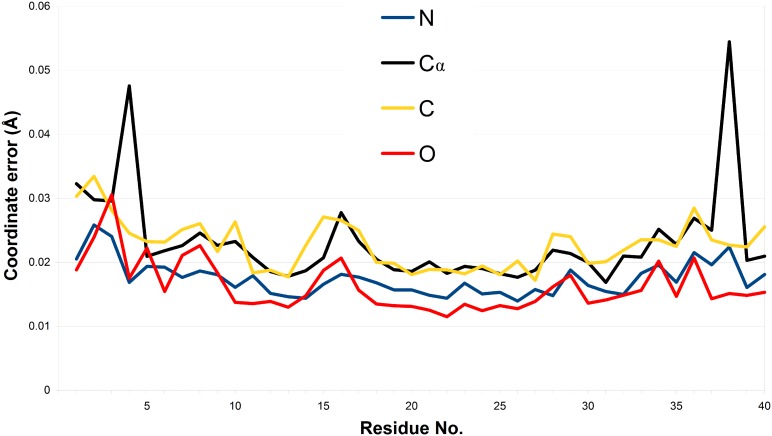
Coordinate errors for main-chain atoms estimated from the inversion of the least-squares matrix. For dual-occupancy atoms, only the major component is plotted.

The estimated values of s.u. in all positional parameters for the fully occupied main-chain atoms range from 0.012 Å to 0.033 Å (Fig. 4). It is evident from Fig. 4 that coordinate errors have smaller values for ‘heavier’ atoms, e.g. oxygen, and slightly higher for nitrogen and carbon atoms. The s.u. values for the major conformation of the two main-chain atoms refined in two conformations are much higher, 0.048 Å for the C_α_ atom of Ala4 and 0.054 Å for the Glu38. A similar pattern was observed for the coordinate errors estimated for the structure of lysozyme refined at 0.65 Å resolution [37] or BPTI refined at 0.86 Å [38]. The quality of the crystallographic model can also be assessed using the statistics of the derived geometrical parameters. For instance, the peptide C = O bond lengths (range from 1.19 to 1.28 Å, with a mean of 1.23 Å) are characterized by standard uncertainties between 0.01 and 0.03 Å, with a mean of 0.02 Å. These statistics are almost identical to those reported for squash trypsin inhibitor (CMTI-I) studied at a comparable resolution of 1.03 Å [39]. However, the model of CMTI-I was refined with the BUMP and geometrical restraints retained on main-chain segments with excessive displacement parameters (with *B*_eq_>15 Å).

### Crystal packing and intermolecular contacts

**Figure 5:**
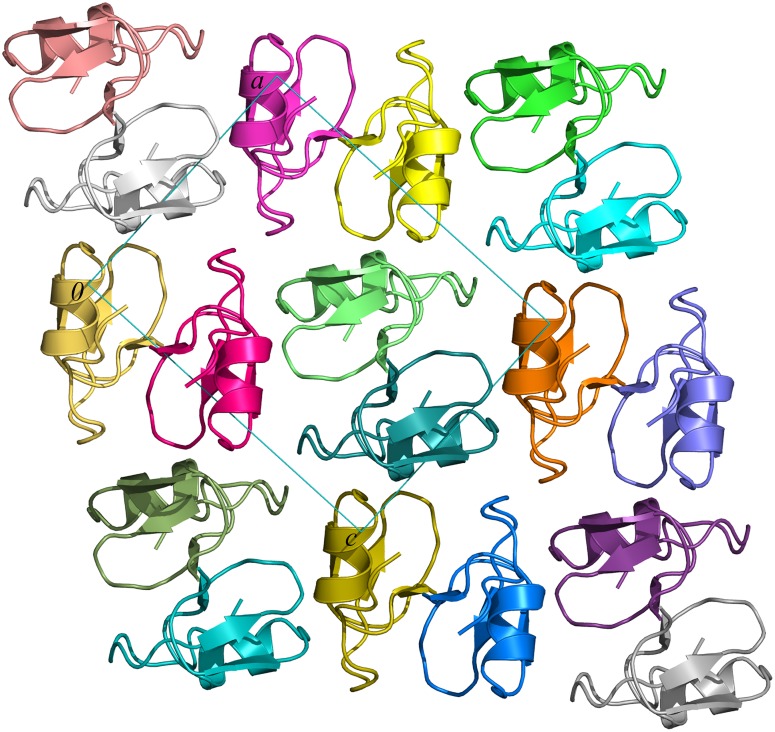
Crystal packing of *Gm*SPI-2 molecules viewed down the crystallographic *b* axis.

The *Gm*SPI-2 molecules are densely packed with only 30.2% volume being occupied by solvent (the corresponding Matthews coefficient is 1.76 Å^3^/Da). All other Kazal-family serine proteinase inhibitors are more loosely packed, with the solvent content ranging from 31.9% (for N-terminally truncated turkey ovomucoid third domain, OMTKY3; PDB code 2 gkr) to 54.0% (for infestin 4; 2erw). The *Gm*SPI-2 molecules are more solvent exposed along the crystallographic *b* axis (Fig. 5). There are 7 intermolecular hydrogen bonds, listed in Table 3. Two of them, linking molecules related by the 2_1_ screw along [001] involve atoms with partial occupancy. The hydrogen bond involving the Cys24 N atom should be regarded as week due to an unfavorable angle and the presence of another hydrogen acceptor from the preceding Asn22 O_δ1_ atom. Besides direct hydrogen bonds, water molecules play a profound role in mediating intermolecular contacts. An example of this is the N-terminus where the Glu1 N atom is anchored to two symmetry related *Gm*SPI-2 molecules by hydrogen bonds through three well-ordered water molecules 203, 204 and 226 with *B*-factors (Å^2^)/donor-acceptor distances (Å) of 7.30/2.85, 7.99/2.82 and 6.27/2.70, respectively. Moreover, the first three N-terminal residues, which in fact are artificial to the native *Gm*SPI-2 sequence, form only indirect intermolecular interactions through water molecules and a sodium ion. The first direct intermolecular hydrogen bond is made by the Val4 O atom (Table 3).

**Table 3:** Direct protein-protein intermolecular hydrogen bonds in the *Gm*SPI2 crystal structure.

No	Residue, atom	Residue, atom	Distance(Å)
1	Val4, O	Gly32(a), N	2.83
2	Thr6, N	Gly30(a), O	2.90
3	Cys24, N	Ala29(a), O	3.06
4	Ser21, N	Leu40(b), OXT	2.77
5	Ser21, O_γ_	Leu40(b), O	2.58
6	Glu8, O_ε2_	Lys15(c), N_ζ_	2.42
7	Asn27, N_δ2_	Glu38(c), O_ε1_	3.07

(a) x +½, −y + ½, −z.

(b) −x, y + ½, −z+½.

(c) −x+½, −y, z+½.

The crystal structure includes four partially occupied sodium ions. Two of them, namely Na101 and Na102 have complete octahedral coordination spheres. The coordination number of Na103 and Na104 is 5 and 4, respectively. These sodium ions are coordinated by oxygen atoms belonging to the *Gm*SPI-2 molecule (Thr7 O_γ1_, Asp16 O and Asp34 O and O_δ1_), as well as by water molecules. It has not escaped our notice that two sodium ions, namely Na103 and symmetry related Na104 (−x, ½+y, ½−z), are 3.14 Å apart sharing two symmetry related water molecules 257 and 258 (−x, ½+y, ½−z) in their coordination spheres. Similar arrangement is often found in small molecule structures. In the structure of catena-(hexakis(µ_2_-Aqua)-di-sodium 2,5-dibenzoylterephthalate tetrahydrate) (CSD reference code HAYQUU) [40] Na1 and Na2 ions are 3.14 Å apart and share three water molecules (O4, O5 and O6) in their coordination spheres.

### Description of the *Gm*SPI2 structure

**Figure 6:**
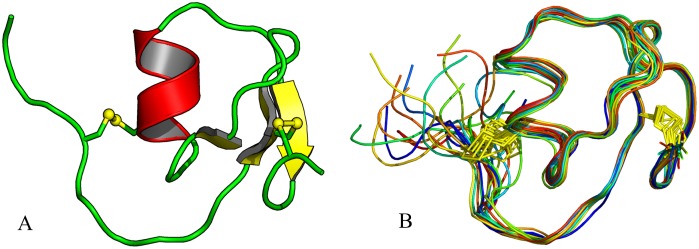
The overall X-ray (A) and NMR (B) models of *Gm*SPI-2. (A) β-stands and disulfides are shown in yellow, α-helix in red and loops in green. (B) The ensemble of 17 lowest energy conformers are shown in different colors, with disulfides in yellow. The figure was prepared using *PyMOL* [47].

**Figure 7:**
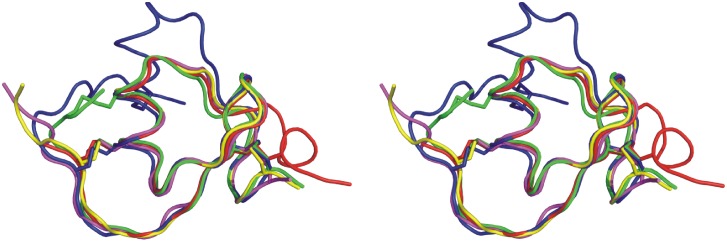
Stereo C_α_ tracing of the crystallographic model of *Gm*SPI-2 (yellow) superimposed with the NMR structure of *Gm*SPI-2 in solution (conformer 11, magenta), *Cr*SPI-1-D1 (red, PDB code 3pis), OMTKY3 (blue, 2 gkr) and LDTI (green, 1an1). The superposition was calculated in *Coot* (Emsley & Cowtan, 2004) using the *SSM* algorithm and displayed in *PyMOL* [47].

The overall structure of *Gm*SPI-2 (Fig. 6A) resembles that of other Kazal-family serine proteinase inhibitors. Residues Val12-Gly14, Thr19-Tyr20 and Leu33-Glu36 form an anti-parallel β-sheet while residues Leu23-Ala29 form the central α-helix of the characteristic Kazal-family serine proteinase inhibitor β_2_αβ fold. *Gm*SPI-2 shows, however, features of non-classical Kazal-family serine proteinase inhibitors, harboring an unusual pattern of disulfide bridges. Only two intradomain disulfide bridges formed between Cys residues 5 and 24 and Cys residues 13 and 39 are present. *Gm*SPI-2 has been classified as a non-classical Kazal-family serine proteinase inhibitor group 1 [41]. This group of inhibitors is characterized by the shift of the first and the fifth half-cystine residues towards the C-terminus with respect to classical Kazal-family serine proteinase inhibitors [42]. A superposition of the X-ray structure of *Gm*SPI-2 with a classical Kazal-family serine proteinase inhibitor (OMTKY3, 2 gkr), a group 1 non-classical Kazal-family serine proteinase inhibitor (*Cr*SPI-1-D1, 3pis) and a group 2 non-classical Kazal-family serine proteinase inhibitor (LDTI, 1an1) shows that it resembles the structure of LDTI (Fig. 7) with a root-mean-square deviation (rmsd) of 0.92 Å for 37 superimposed C_α_ atoms [43]. *Cr*SPI-1-D1 like *Gm*SPI-2 has only two disulfide bridges. Both structures are similar up to Asp34 of *Gm*SPI-2, but very different from there, till the very C-terminus. Residues Trp34-Cys37 of *Cr*SPI-1-D1 form a 3_10_-helix, which is not present in the structure of *Gm*SPI-2.

The fully exposed reactive site loop (RSL) of the inhibitor presents the P1 site with the Thr7 residue. The size of the *Gm*SPI-2 RSL is typical for Kazal-family serine proteinase inhibitors, with seven amino acids between Cys5 and Cys13. It is generally thought that the rigidity of the RSL together with its specific sequence are the key factors conferring high potency on Kazal-family serine proteinase inhibitors [41]. There are usually eight hydrogen bonds stabilizing the RSL of Kazal-family serine proteinase inhibitors [25], [44]. The *Gm*SPI-2 RSL has an additional strong (2.86 Å) hydrogen bond stabilizing the RSL between Thr6 (O) and Trp25 (N_ε_). Amongst Kazal-family serine proteinase inhibitors a tryptophan residue in this position is present only in *Gm*SPI-2 [45].

**Figure 8:**
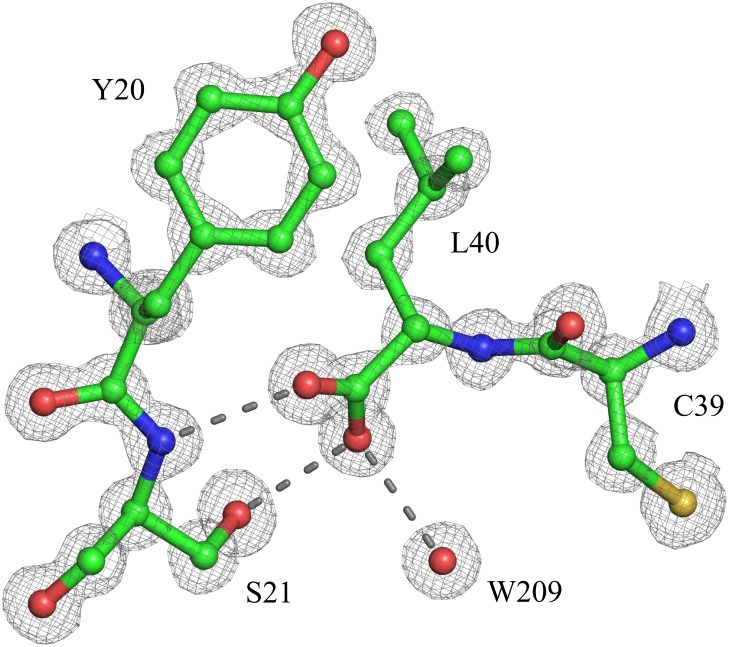
The C-terminus of the crystallographic model of *Gm*SPI-2, residues Cys39 and Leu40, and symmetry-related Tyr20 and Ser21 residues. The 2*F*_o_-*F*_c_ electron density map was contoured at 1.4 σ. Hydrogen bonds are shown as dash lines.

The His-tag cleavage site was located between Leu40, and Ser41 of the fusion protein. The 2*F*_o_-*F*_c_ electron density map for Leu40 is excellent for both the main-chain and side-chain atoms including the C-terminal oxygen atoms O and OXT (Fig. 8). The C-terminus is anchored by three strong hydrogen bonds involving both carboxylic oxygen atoms. Two of them are listed in Table 3. The third one involves the Leu40 OXT and Wat209 (x, y-1, z) atoms. This further confirms the sequence specificity of the presented tag cleavage procedure.

### The structure of the *Gm*SPI-2 protein in solution determined by nmr spectroscopy

The three-dimensional structure of *Gm*SPI-2 in solution was determined by NMR spectroscopy using 503 distance constraints derived from the analysis of 2D 1H-1H NOESY data sets. The ensemble of 17 lowest-energy structures (PDB code 2m5x) selected from a total of 200 calculated models is characterized by good convergence and low rmsd from ideal geometry (Table 2). The NMR-evaluated 3D structure is similar to that determined by X-ray crystallography, and contains the same α (Leu23-Ala29) and β (Val12-Gly14, Thr1-Tyr20, Leu33-Glu36) elements folded into the β2αβ motif of the hydrophobic core of the protein (Fig. 6B). A least-squares superposition of the 34 C_α_ atoms of the Cys5-Glu38 segment of the crystallographic model and the NMR conformer 11, representing the NMR ensemble, is characterized by an rmsd of 0.89 Å. Upon detailed inspection, the indole group of Trp25 and the imidazole ring of His35 are found to be in different orientations in the two models. Specifically, the χ2 torsion angle of Trp25 differs by 30° between the X-ray and NMR structures. In effect, the Trp25 N_ε_⋅ ⋅ ⋅Thr6 O hydrogen bond observed in the crystal structure is not present in solution. Likewise, the His35 H_δ1_⋅ ⋅ ⋅ Glu36 O hydrogen bond is broken in solution due to reorientation of the His35 ring from χ1 of −40° (*gauche*−) to +60° (*gauche*+). Some minor conformational differences observed at the N-termini can be attributed to crystallographic packing.

### Methodological remarks

The applicability of our method is based on the assumption that Ni(II) ions can interact with -SRHW-like sequences only at solvent exposed tags. Otherwise we would be posed with unspecific protein cleavage by Ni(II) ions. In previous study we verified the crucial assumption that Ni(II) ions would not be able to penetrate protein interiors or distort secondary structure elements using human ubiquitin. This protein naturally possesses the potentially active Thr-Leu-His-Leu sequence. However, despite prolonged incubations in the presence of Ni(II) ions at elevated temperatures no cleavage was observed for the natively folded protein, while Ubi denaturated by GuHCl was hydrolyzed [14].

The results obtained by X-ray crystallography and NMR spectroscopy consistently demonstrate that the molecular structure of *Gm*SPI-2 is highly similar in solution and in the crystalline state. However, several small differences could be still detected. For instance, the exact conformation of the two disulfide bridges could not be unambiguously determined from the NMR data due to insufficient concentration of the *Gm*SPI-2 protein in solution. The conformation of the first four N-terminal residues is different in the two structures as a result of crystal packing interactions, *i.e.* absence of intermolecular hydrogen bonds in solution.

His-tag and other small affinity tags are routinely used to obtain pure recombinant proteins, and structural studies in solution are often conducted without tag removal. This is, however, often impossible in the solid state because the crystal packing can lead to non-native interactions between the tag and the rest of the molecule. Therefore, the quality of X-ray data strongly depends on the homogeneity of the protein material, that is on the efficacy of the tag removal procedure and on the absence of non-specific cleavage products, which are usually generated by proteolytic enzymes. In this perspective, the high resolution of the X-ray diffraction data obtained in this work can be related to the truly perfect removal of the affinity tag afforded by the nickel-based methodology. Furthermore, the high yield of this method on the preparative scale (conversion of 70% of the starting material to the final product, with 100% homogeneity) makes it a good tool for obtaining pure thermostable proteins for structural studies.

The short *Gm*SPI-2 gene is a promising target for mutagenesis directed toward engineering novel variants of the protein, specific for selected serine proteinases (study in progress). Such a study must be based on the precise knowledge of the starting polypeptide structure. Only with such knowledge one can carry out rational modeling and docking studies of *Gm*SPI-2 derivatives to identify suitable hits for overexpression and activity evaluation. A clear understanding of the relation between the polypeptide structures in the crystal and in solution is also a prerequisite for the validity of such an approach. In this perspective, the accurate structure of this unique polypeptide belonging to non-classical Kazal-family serine proteinase inhibitors, by two methods, has to be regarded as setting the stage for further studies. It is worth mentioning that in a set of biologically interesting proteins, short domains with varying homology to *Gm*SPI-2 seem to be frequently present at either the N- or the C-terminus (Kaczanowski & Zagórski, unpublished). Prediction of the probable function of such domains will be facilitated by the present results, which have defined the structural properties of a bona-fide inhibitor.

## Conclusions

In the present study, the *Gm*SPI-2 protein sequence was extended C-terminally by an -SRHWAP-H_6_ dodecapeptide, which comprises the underlined Ni(II)-sensitive tetrapeptide linked to the His-tag domain. The fusion protein was expressed in *Pichia pastoris* and affinity purified on Ni-NTA columns. The cleavage of the tag directly on the Ni-NTA column enabled us to combine the affinity purification and the tag removal into one step. The *Gm*SPI-2 protein obtained in flow-through fractions exhibited 100% homogeneity. The absolute sequence specificity of the cleavage, observed previously in analytical scale purifications, has been preserved on the preparative scale as well. No protein impurities whatsoever could be detected in the protein fractions tested by HPLC and ESI-MS. The efficiency of cleavage was 70% on the preparative scale. The resulting *Gm*SPI-2 protein was fully active. The results obtained by X-ray crystallography and NMR spectroscopy show that the structure of *Gm*SPI-2 is highly similar in solution and in the crystalline state. The resolution of the crystal structure of 0.98 Å is the highest for the Kazal-type serine protease inhibitors deposited in the PDB. The number of reflections per parameter justified refinement without any stereochemical restraints for the well-ordered parts of the inhibitor. The refinement converged with *R* = 10.57% for all reflections. One cycle of full-matrix minimization permitted the estimation of the standard uncertainties in all positional parameters which, for example, for the fully occupied main-chain atoms range from 0.012 Å to 0.033 Å. The 2*F*_o_-*F*_c_ electron density map for Leu40, the last residue of the mature inhibitor, is excellent for both the main-chain and side-chain atoms including the C-terminal oxygen atoms O and OXT. This clearly confirms the sequence specificity of the presented tag cleavage procedure.

These exceptionally high grade of the protein purification product was reflected in the high quality of the structural determinations, both in the solid state and in solution. The high resolution of these structures was certainly facilitated by the perfect homogeneity of the protein sample after affinity tag removal.
